# Association of hemoglobin A1c with the incidence of hypertension: A large prospective study

**DOI:** 10.3389/fendo.2022.1098012

**Published:** 2023-01-16

**Authors:** Xu Huang, Cheng Qin, Xiaoxu Guo, Feng Cao, Chengchun Tang

**Affiliations:** ^1^ Department of Cardiology, Zhongda Hospital, School of Medicine, Southeast University, Nanjing, China; ^2^ Department of Geriatric Cardiology, National Clinical Research Center for Geriatric Diseases, 2nd Medical Center, Chinese PLA General Hospital, Beijing, China; ^3^ Department of Digestive Diseases, Beijing Luhe Hospital, Capital Medical University, Beijing, China

**Keywords:** glycated hemoglobin, hemoglobin A1c, hypertension, prevalence, incidence

## Abstract

**Background:**

Although hemoglobin A1c (HbA1c) is closely related to diabetes, its relationship with the incidence of hypertension is still unknown, so we aimed to evaluate the relationship between HbA1c and the incidence of hypertension in the general population.

**Method:**

In this large prospective cohort study with a median follow-up of 2 years, we included 4,074 participants from the China Health and Nutrition Survey (CHNS). Multivariate COX regression, subgroup analysis, receiver operator characteristic (ROC) curve and restricted cubic spline (RCS) were used to evaluate the relationship between HbA1c and incidental hypertension.

**Results:**

Compared with participants without incident hypertension, participants with incident hypertension had higher levels of HbA1c (P < 0.05). In univariate COX regression analysis, HbA1c was associated with the risk of hypertension (HR: 1.161, 95% CI: 1.105-1.221, P < 0.001). In multivariate COX regression analysis adjusted for confounding variables, HbA1c was still closely related to the risk of hypertension (HR: 1.102, 95% CI: 1.006-1.206, P = 0.037). And subgroup analysis showed that the relationship between HbA1c and hypertension remained significant in female, lower than high school and non-obese subgroups (P < 0.05). ROC curve also showed that HbA1c could predict the risk of hypertension (AUC = 0.583, 95% CI: 0.568-0.598, P < 0.001). Further RCS analysis showed that HbA1c was positively correlated with the risk of hypertension (P for nonlinearity = 0.642).

**Conclusion:**

HbA1c was linearly and positively associated with the incidence of hypertension.

## 1 Introduction

Currently, arterial hypertension (hereinafter referred to as hypertension) is a very common disease and one of the most important risk factors for cardiovascular disease and premature death (1). In 2 years, it is expected that more than 1.5 billion people will suffer from hypertension, which undoubtedly indicates that hypertension has gradually become a major global public health problem (2). The reasons for the dramatic increase in hypertension stem not only from an aging population, unhealthy lifestyles and unhealthy diets, but also from other metabolic problems, such as fluctuating blood glucose or diabetes (3).

Similar to hypertension, at present, diabetes is also a major global public health problem. In 2019, nearly 463 million people developed diabetes (9.3%), and this percentage is expected to rise by 0.9% and 1.6% by 2030 and 2045, respectively (4). As two major risk factors for cardiovascular disease and mortality, hypertension and diabetes tend to coexist in the same metabolically dysregulated individual and they share some common abnormal metabolic pathways, such as obesity, insulin resistance, inflammation and oxidative stress (5–7). Current evidence has shown that diabetes is closely related to hypertension (8, 9), while it is uncertain whether blood glucose fluctuations are associated with hypertension. Hemoglobin A1c (HbA1c) is not only one of the most important tools for diagnosing diabetes superior to fasting blood glucose, but also an indicator of blood glucose fluctuations and the efficacy of glycemic control over the last 3 months (10, 11). Although the relationship between HbA1c and cardiovascular disease and mortality has been reported in many studies (12–14), the research on its relationship with the prevalence and incidence of hypertension is still few and not unified. As mentioned earlier, the harm of hypertension and diabetes is great, so it is necessary to control the incidence of hypertension and diabetes to reduce the socio-economic burden and public health. HbA1c is not only a diagnostic factor of diabetes, but also has been proved to be closely related to cardiovascular disease and mortality. Assuming that there is a causal relationship between HbA1c and the incidence of hypertension, then controlling the level of HbA1c not only reduces the incidence of hypertension but also reduces the burden of diabetes, which is not only a treatment of killing two birds with one stone, but also has far-reaching significance in reducing the economic burden, the incidence of metabolic-related diseases and the reduction of premature death.

Therefore, in order to enrich this research area and provide more evidence for evidence-based medicine, this study aimed to explore the relationship between HbA1c and the incidence of hypertension in a general population in the Chinese community.

## 2 Subjects, materials and methods

### 2.1 Study population

**Figure 1:**
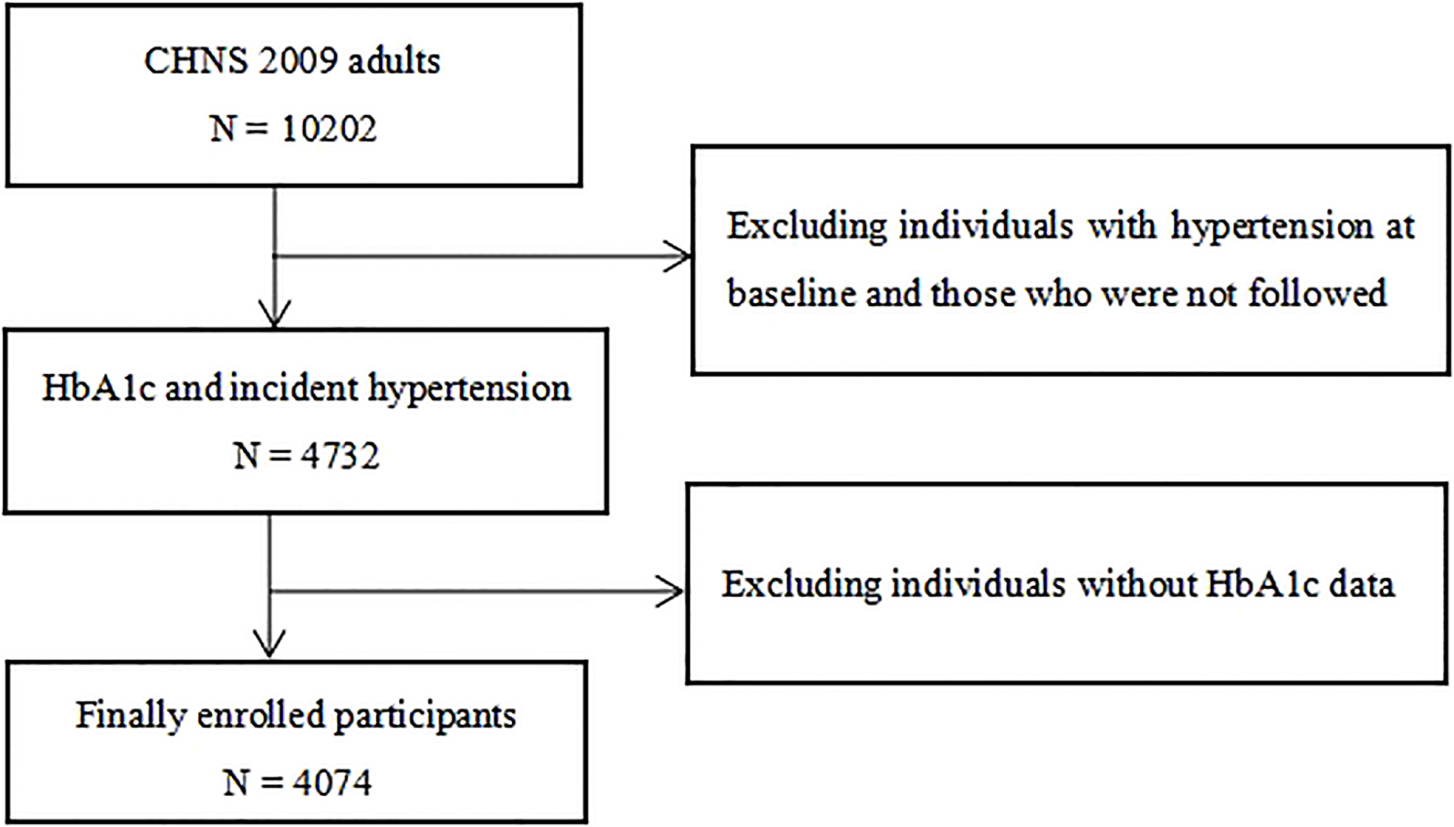
Flow chart of the study population. CHNS, China Health and Nutrition Survey; HbA1c, hemoglobin A1c.

This was a large prospective cohort study based on community populations, with all participants from the 2009 China Health and Nutrition Survey (CHNS 2009). After excluding individuals with baseline hypertension and those without HbA1c and follow-up data, a total of 4,074 individuals were enrolled in the study (**Figure 1**). The CHNS was approved by the institutional review committees at the University of North Carolina at Chapel Hill and the National Institute of Nutrition and Food Safety, Chinese Center for Disease Control and Prevention. Every participant signed a written informed consent form when participating in the CHNS, and the study protocol was carried out in accordance with the Declaration of Helsinki.

### 2.2 Data collection and definitions

All the data included in this study were from CHNS 2009, including demographic data, complications data, drug treatment data, biomarker data and follow-up data, in which the educational level was divided into three groups: lower than high school, high school and higher than high school. Marital status was divided into two groups: married and non-married. Smoking status was divided into three groups: now, ever and never. Drinking status was divided into five groups: every day, 3-4 times/week, 1-2 times/week, ≤ 2 times/month and no drinking (15). Diabetes was defined as fasting blood glucose ≥ 7.0 mmol/L, HbA1c ≥ 6.5%, or using hypoglycemic drugs, or having a history of diabetes diagnosis (16). Incidental hypertension was defined as newly diagnosed hypertension when non-hypertensive individuals participating in CHNS 2009 re-participated in CHNS 2011 and 2015 by asking for medical history and blood pressure measurements, such as systolic blood pressure (SBP) and/or diastolic blood pressure (DBP) ≥ 140/90 mmHg. Anthropometric data, including body mass index (BMI), SBP, DBP, were measured by trained staff from CHNS in accordance with standard measurement procedures. Blood markers, including triglycerides (TG), total cholesterol (TC), low-density lipoprotein cholesterol (LDL-C), high-density lipoprotein cholesterol (HDL-C), apolipoprotein A1 (ApoA1), apolipoprotein B (ApoB), creatinine (CR), fasting plasma glucose (FPG), HbA1c and high-sensitivity C-reactive protein (Hs-CRP), were collected by trained CHNS staff and sent to a standard laboratory for determination according to standard operating procedures, of which HbA1c was determined by high performance liquid chromatography (model HLC-723 G7; Tosoh Corporation, Tokyo, Japan), and the levels of FPG, blood lipids and Hs-CRP were measured by GODPAP method (Randox Laboratories Ltd., UK), glycerol-phosphate oxidase method and the PEG-modified enzyme method (Kyowa Medex Co., Ltd, Tokyo, Japan), and immunoturbidimetric method (Hitachi 7600 automated analyzer, Hitachi Inc., Tokyo, Japan) respectively (17).

### 2.3 Statistical analysis

The continuous variables with normal or skewed distribution were expressed by mean ± standard deviation or median (first quartile, third quartile), respectively, and the differences between groups were tested by independent sample T test or Mann-Whitney U test. The classification variables were presented by frequency (percentage), and the differences between groups were compared by chi-square test and Fisher’s exact test. Univariate COX regression analysis was used to evaluate the relationship between each variable and the incidence of hypertension, and then the covariates with P < 0.05 and significant variables were selected to construct a multivariate COX proportional hazard regression model to evaluate the relationship between HbA1c and the incidence of hypertension. Subgroup analysis based on age, sex, educational level, diabetes, and obesity was used to evaluate the relationship between HbA1c and the incidence of hypertension in these subgroups and the potential interaction between HbA1c and these stratified variables. Receiver operator characteristic (ROC) curve was used to evaluate the ability of HbA1c to distinguish hypertension. Restricted cubic spline (RCS) was used to explore the potential nonlinear association between HbA1c and the risk of hypertension. Using SPSS 26.0, MedCalc 19.6.1 and R 3.6.3 for statistical analysis. A two-tailed P value < 0.05 was determined to be statistically significant.

## 3 Results

### 3.1 Baseline characteristics of study population

As shown in **Table 1**, participants with incident hypertension had higher age, higher rates of education below high school, current smoking, daily alcohol consumption, diabetes, and hypoglycemic drugs use, and higher levels of BMI, SBP, DBP, TG, TC, LDL-C, ApoB, uric acid, FPG, Hs-CRP, and HbA1c compared with participants without incident hypertension (P < 0.05). However, there was no significant difference in marital status, HDL-C, ApoA1 and CR between the two groups (P > 0.05).

**Table 1:** Baseline characteristics of participants stratified by the hypertension.

	Total population	Non-hypertension	Hypertension	P value
Age, years	48.25 ± 13.14	45.80 ± 12.82	53.13 ± 12.39	< 0.001
Sex, male, n (%)	1868 (45.90%)	1195 (44.10%)	673 (49.40%)	0.001
Educational level, n (%)				< 0.001
Lower than high school	3139 (77.00%)	2037 (75.10%)	1102 (80.90%)	
High School	499 (12.20%)	350 (12.90%)	149 (10.90%)	
Higher than high school	431 (10.60%)	321 (11.80%)	110 (8.10%)	
Marital status, n (%)				0.769
Married	3630 (89.10%)	2411 (88.90%)	1219 (89.50%)	
Non-married	436 (10.70%)	295 (10.90%)	141 (10.40%)	
Smoking status, n (%)				0.035
Now	1170 (28.70%)	750 (27.70%)	420 (30.80%)	
Ever	98 (2.40%)	58 (2.10%)	40 (2.90%)	
Never	2804 (68.80%)	1902 (70.10%)	902 (66.20%)	
Drinking status, n (%)				< 0.001
Every day	369 (9.10%)	205 (7.60%)	164 (12.00%)	
3-4 times/week	181 (4.40%)	110 (4.10%)	71 (5.20%)	
1-2 times/week	325 (8.00%)	203 (7.50%)	122 (9.00%)	
≤ 2 times/month	475 (11.70%)	345 (12.70%)	130 (9.50%)	
No drinking	2724 (66.90%)	1849 (68.20%)	875 (64.20%)	
Diabetes, n (%)	309 (7.60%)	168 (6.20%)	141 (10.40%)	< 0.001
Hypoglycemic drugs, n (%)	47 (1.20%)	21 (0.80%)	26 (1.90%)	0.004
BMI, kg/m^2^	22.98 ± 3.23	22.58 ± 3.09	23.78 ± 3.35	< 0.001
SBP, mmHg	116.60 ± 11.23	114.45 ± 11.03	121.05 ± 10.30	< 0.001
DBP, mmHg	76.01 ± 7.46	74.89 ± 7.51	78.35 ± 6.78	< 0.001
TG, mmol/L	1.19 (0.81, 1.81)	1.13 (0.78, 1.72)	1.33 (0.91, 2.02)	< 0.001
TC, mmol/L	4.80 ± 0.96	4.73 ± 0.93	4.95 ± 1.01	< 0.001
LDL−C, mmol/L	2.93 ± 0.91	2.87 ± 0.86	3.05 ± 1.01	< 0.001
HDL−C, mmol/L	1.45 ± 0.46	1.45 ± 0.47	1.44 ± 0.44	0.442
ApoA1, g/L	1.15 ± 0.37	1.15 ± 0.35	1.17 ± 0.41	0.074
ApoB, g/L	0.89 ± 0.25	0.87 ± 0.24	0.93 ± 0.26	< 0.001
CR, umol/L	86.20 ± 21.32	85.83 ± 23.52	86.94 ± 16.05	0.118
Uric acid, umol/L	299.12 ± 100.95	294.89 ± 103.64	307.54 ± 94.82	< 0.001
FPG, mmol/L	5.28 ± 1.29	5.18 ± 1.15	5.46 ± 1.51	< 0.001
HbA1c, %	5.54 ± 0.78	5.47 ± 0.68	5.68 ± 0.94	< 0.001
Hs-CRP, mg/L	1.00 (0, 2.00)	1.00 (0, 2.00)	1.00 (1.00, 2.75)	< 0.001
Follow-up time, years	2.00 (2.00, 6.00)	2.00 (2.00, 2.00)	6.00 (2.00, 6.00)	< 0.001

Data were expressed as mean ± SD, median (interquartile range), or n (%). BMI, body mass index; SBP, systolic blood pressure; DBP, diastolic blood pressure; TG, triglycerides; TC, total cholesterol; LDL-C, low-density lipoprotein cholesterol; HDL-C, high-density lipoprotein cholesterol; ApoA1, apolipoprotein A1; ApoB, apolipoprotein B; CR, creatinine; FPG, fasting plasma glucose; HbA1c, hemoglobin A1c; Hs-CRP, high-sensitivity C-reactive protein.

### 3.2 Association of HbA1c with the incidence of hypertension

**Figure 2:**
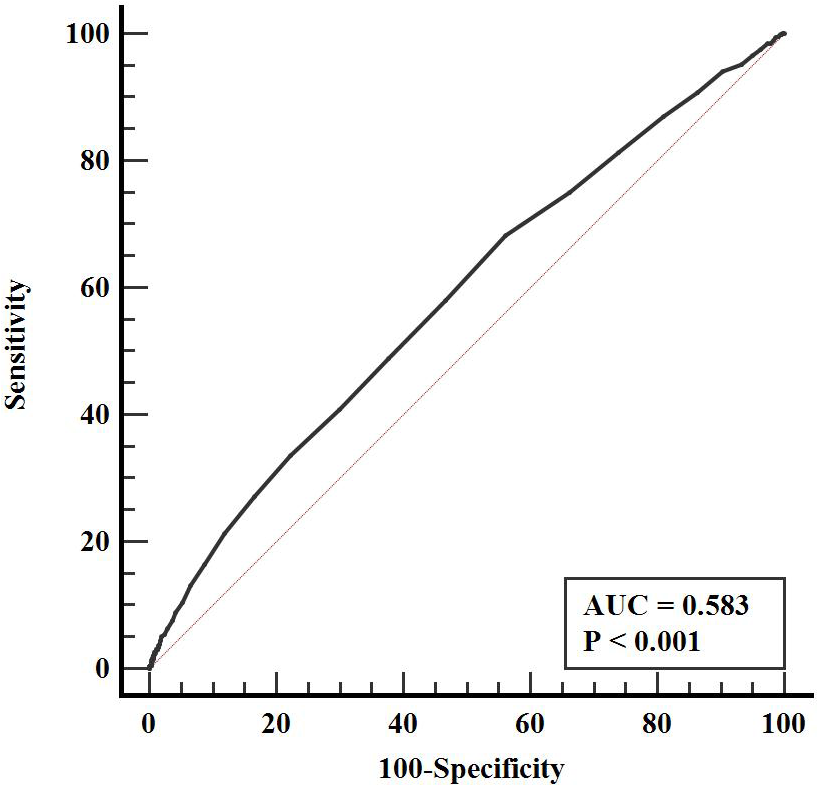
ROC curve evaluating diagnostic performance of HbA1c for incident hypertension. HbA1c, hemoglobin A1c; ROC, receiver operator characteristic; AUC, area under the curve.

**Figure 3:**
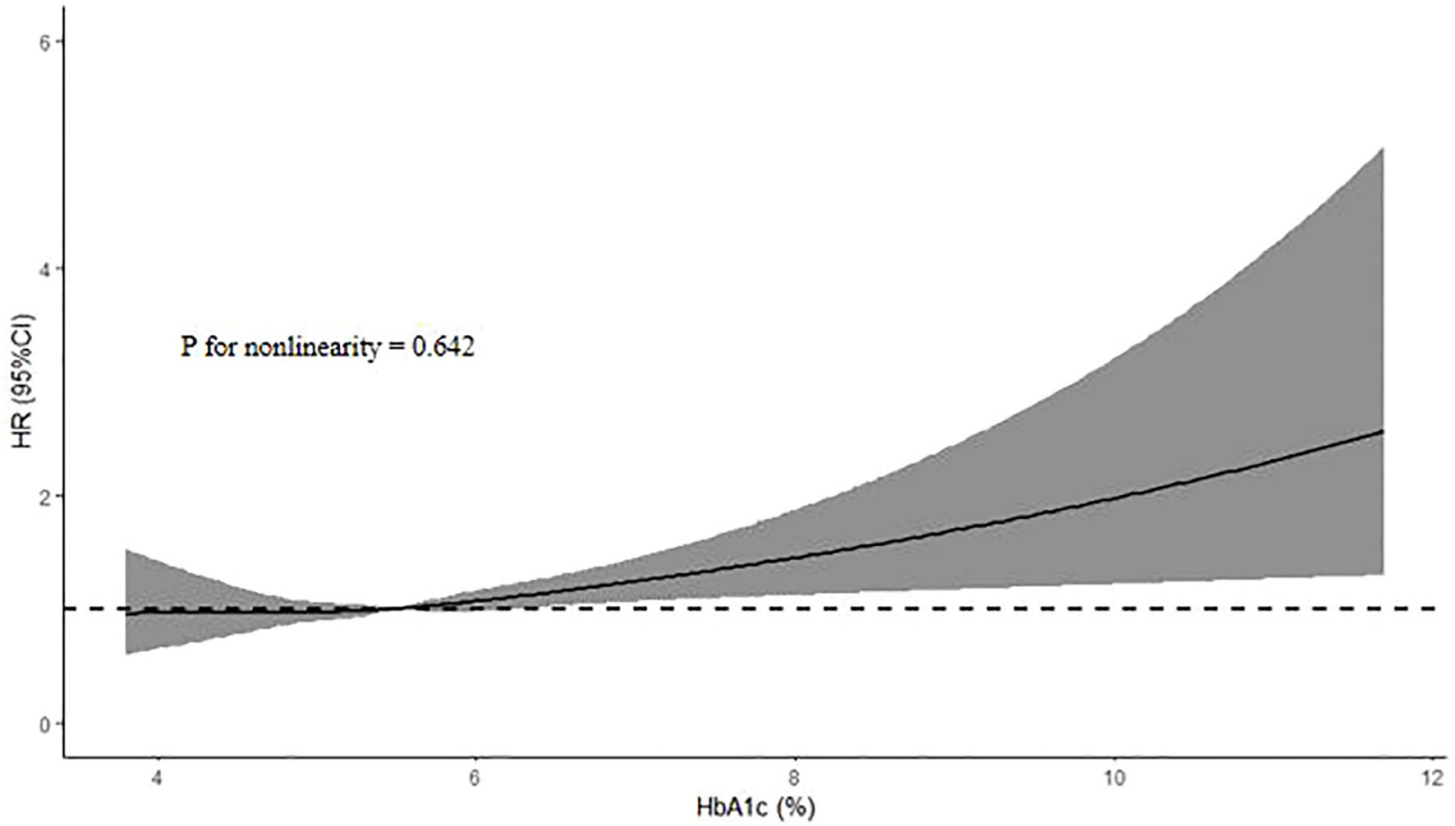
HR (95% CI) for the incidence of hypertension according to HbA1c. The association was adjusted for variables included in the multivariate adjusted models in **Table 2**. HbA1c, hemoglobin A1c; HR, hazard ratio; CI, confidence interval.

As shown in **Table 2**, HbA1c was associated with the risk of hypertension in univariate COX regression analysis (HR: 1.161, 95% CI: 1.105-1.221, P < 0.001), and age, sex, educational level, smoking status, drinking status, diabetes, hypoglycemic drugs, BMI, SBP, DBP, TG, TC, LDL-C, ApoB, CR, uric acid, FPG and Hs-CRP were also associated with the risk of hypertension (P < 0.05). In multivariate COX regression analysis, higher HbA1c was still associated with higher risk of hypertension after adjusting for age, sex, educational level, marital status, smoking status, drinking status, diabetes, hypoglycemic drugs, BMI, SBP, DBP, TG, TC, LDL-C, ApoB, CR, uric acid, FPG and Hs-CRP (HR: 1.102, 95% CI: 1.006-1.206, P = 0.037). And in the subgroup analysis of **Table 3**, the association was still significant in women, lower than high school and non-obese subgroups (HR: 1.158, 95% CI: 1.007-1.331, P = 0.039; HR: 1.127, 95% CI: 1.022-1.243, P = 0.017; HR: 1.073, 95% CI: 1.007-1.142, P = 0.029; respectively), whlie the relationship between HbA1c and new-onset hypertension no longer existed in the subgroups of < 60 years, ≥ 60 years, male, high school, higher than high school, diabetes, non-diabetes and obesity (P > 0.05). In addition, ROC analysis showed that HbA1c could predict the occurrence of hypertension (AUC = 0.583, 95% CI: 0.568-0.598, P < 0.001) (**Figure 2**). Further RCS analysis showed that there was a positive linear correlation between HbA1c and the risk of hypertension (P for nonlinearity = 0.642) (**Figure 3**). In addition, as shown in **Table 4**, we conducted a sensitivity analysis showing that higher HbA1c was still associated with a higher risk of hypertension (P < 0.05).

**Table 2:** Univariate and multivariate COX regression analysis of incident hypertension.

	Univariate		Multivariate	
	HR (95% CI)	P value	HR (95% CI)	P value
Age	1.025 (1.020, 1.029)	< 0.001	1.022 (1.017, 1.027)	< 0.001
Male	1.135 (1.021, 1.263)	0.019	1.081 (0.914, 1.279)	0.361
Higher than high school	0.737 (0.606, 0.897)	0.002	0.809 (0.662, 0.989)	0.038
Married	0.964 (0.810, 1.148)	0.684	0.925 (0.774, 1.105)	0.389
Smoking status: Never	0.888 (0.791, 0.997)	0.045	0.941 (0.811, 1.091)	0.420
Drinking status: No drinking	0.776 (0.657, 0.917)	0.003	0.899 (0.743, 1.087)	0.270
Diabetes	1.390 (1.168, 1.655)	< 0.001	0.889 (0.696, 1.135)	0.346
Hypoglycemic drugs	1.712 (1.161, 2.523)	0.007	1.151 (0.738, 1.796)	0.534
BMI	1.048 (1.031, 1.066)	< 0.001	1.039 (1.020, 1.058)	< 0.001
SBP	1.032 (1.026, 1.038)	< 0.001	1.010 (1.004, 1.016)	0.001
DBP	1.035 (1.026, 1.045)	< 0.001	1.009 (0.999, 1.018)	0.064
TG	1.056 (1.022, 1.091)	0.001	1.027 (0.972, 1.084)	0.349
TC	1.115 (1.058, 1.176)	< 0.001	0.982 (0.874, 1.103)	0.754
LDL−C	1.085 (1.030, 1.143)	0.002	0.992 (0.877, 1.123)	0.902
HDL−C	0.971 (0.864, 1.092)	0.626		
ApoA1	1.077 (0.961, 1.208)	0.203		
ApoB	1.610 (1.312, 1.976)	< 0.001	1.133 (0.720, 1.783)	0.588
CR	1.002 (1.000, 1.004)	0.033	1.000 (0.996, 1.004)	0.987
Uric acid	1.001 (1.000, 1.001)	0.007	1.000 (0.999, 1.001)	0.840
FPG	1.075 (1.041, 1.111)	< 0.001	0.984 (0.929, 1.043)	0.590
Hs-CRP	1.010 (1.004, 1.016)	0.002	1.005 (0.997, 1.012)	0.208
HbA1c	1.161 (1.105, 1.221)	< 0.001	1.102 (1.006, 1.206)	0.037

BMI, body mass index; SBP, systolic blood pressure; DBP, diastolic blood pressure; TG, triglycerides; TC, total cholesterol; LDL-C, low-density lipoprotein cholesterol; HDL-C, high-density lipoprotein cholesterol; ApoA1, apolipoprotein A1; ApoB, apolipoprotein B; CR, creatinine; FPG, fasting plasma glucose; Hs-CRP, high-sensitivity C-reactive protein; HbA1c, hemoglobin A1c; HR, hazard ratio; CI, confidence interval.

**Table 3:** Subgroups analyses for the association between HbA1c and the incidence of hypertension.

	HR (95% CI)	P value	P for interaction
Age			0.270
< 60 years	1.082 (0.961, 1.217)	0.191	
≥ 60 years	1.139 (0.979, 1.324)	0.092	
Sex			0.672
Male	1.062 (0.938, 1.201)	0.342	
Female	1.158 (1.007, 1.331)	0.039	
Educational level			0.901
Lower than high school	1.127 (1.022, 1.243)	0.017	
High School	0.981 (0.671, 1.434)	0.921	
Higher than high school	0.922 (0.627, 1.355)	0.678	
Diabetes			0.925
Yes	1.119 (0.972, 1.290)	0.118	
No	1.096 (0.954, 1.259)	0.197	
Obesity			0.423
Yes	1.307 (0.881, 1.938)	0.183	
No	1.073 (1.007, 1.142)	0.029	

The multivariate adjusted model used in the subgroups analysis consisted of all covariates used in the multivariate adjusted models in **Table 2** except for the variable (as a categorical variable) that was used for stratification. The HR was examined by per 1-unit increase of HbA1c. The interaction of HbA1c and variables used for stratification was examined by likelihood ratio tests. HbA1c, hemoglobin A1c; HR, hazard ratio; CI, confidence interval.

**Table 4:** Sensitivity analysis for the association between HbA1c and the incidence of hypertension.

	Model 1		Model 2	
	HR (95% CI)	P value	HR (95% CI)	P value
Age	1.022 (1.018, 1.027)	< 0.001	1.023 (1.019, 1.028)	< 0.001
Male	1.081 (0.929, 1.258)	0.315	1.153 (1.036, 1.285)	0.009
Higher than high school	0.804 (0.658, 0.983)	0.033		
Married	0.927 (0.776, 1.107)	0.401		
Smoking status: Never	0.944 (0.814, 1.095)	0.446		
Drinking status: No drinking	0.893 (0.738, 1.080)	0.242		
Diabetes	0.889 (0.696, 1.135)	0.345	0.899 (0.708, 1.142)	0.382
Hypoglycemic drugs	1.136 (0.729, 1.771)	0.574		
BMI	1.040 (1.022, 1.059)	< 0.001	1.040 (1.022, 1.058)	< 0.001
SBP	1.010 (1.004, 1.016)	0.001	1.010 (1.004, 1.016)	0.001
DBP	1.009 (0.999, 1.018)	0.064	1.009 (1.000, 1.018)	0.059
TG	1.022 (0.973, 1.073)	0.389	1.023 (0.973, 1.074)	0.376
TC	0.981 (0.873, 1.101)	0.743	0.990 (0.881, 1.112)	0.862
LDL−C	0.988 (0.872, 1.119)	0.848	0.986 (0.867, 1.121)	0.832
HDL−C				
ApoA1				
ApoB	1.144 (0.729, 1.795)	0.559	1.102 (0.702, 1.731)	0.674
CR				
Uric acid				
FPG	0.987 (0.932, 1.045)	0.652	0.988 (0.933, 1.046)	0.669
Hs-CRP				
HbA1c	1.100 (1.006, 1.204)	0.037	1.101 (1.007, 1.205)	0.035

BMI, body mass index; SBP, systolic blood pressure; DBP, diastolic blood pressure; TG, triglycerides; TC, total cholesterol; LDL-C, low-density lipoprotein cholesterol; HDL-C, high-density lipoprotein cholesterol; ApoA1, apolipoprotein A1; ApoB, apolipoprotein B; CR, creatinine; FPG, fasting plasma glucose; Hs-CRP, high-sensitivity C-reactive protein; HbA1c, hemoglobin A1c; HR, hazard ratio; CI, confidence interval.

## 4 Discussion

Although there is evidence that HbA1c is associated with hypertension, the relationship between them in people from CHNS is unknown. In this large prospective cohort study, we not only confirmed that HbA1c was closely related to the incidence of hypertension during follow-up, but also confirmed that this significant correlation still existed in women, lower than high school and non-obese subgroups, and further confirmed that there was a linear positive correlation between HbA1c and the risk of hypertension, which not only filled the knowledge gap of CHNS, but also stabilized the stability of the relationship between HbA1c and hypertension in Chinese population.

Although our study had made meaningful findings, there are still few studies on the relationship between HbA1c and hypertension and no unified conclusion has been reached. For example, Britton et al. found in a large prospective cohort study of 19,858 women in 2011 that higher HbA1c was closely associated with the risk of developing hypertension during an average follow-up period of 11.6 years, while this correlation could not be independent of BMI (18). A large longitudinal study from Japan also found no independent association between HbA1c and future new-onset hypertension (19). The evidence from a large medical center also only revealed the relationship between fasting blood glucose and the incidence of hypertension in prediabetes, and did not confirm the independent predictive effect of HbA1c on the incidence of hypertension (20). Similar to the above studies, Tatsumi et al. also only found an independent predictive effect of fasting blood glucose on new-onset hypertension in the cohort from Japan, and failed to confirm the independent correlation between HbA1c and new-onset hypertension (21). A Mendelian randomized study showed that in a univariate linear Mendelian random analysis, each 1 mmol/mol increase in HbA1c predicted by the gene increased the risk of hypertension by 2%, but this correlation no longer existed after adjusting for hemoglobin (22). Besides, another multicenter clinical study from China showed that the higher baseline HbA1c was not an independent risk factor for the incidence of hypertension in the multivariate adjusted model, while the absolute rate of change in HbA1c levels was independently associated with the risk of hypertension (23). However, Omar et al. confirmed a positive correlation between HbA1c levels and the risk of newly diagnosed hypertension in a small cross-sectional study (24). And in a study involving 9,603 middle-aged people, Julie et al. showed that higher HbA1c was not only independently associated with the prevalence of hypertension, but also with the incidence of hypertension (25). And a Mendelian randomized study using the UK Biobank data showed that higher HbA1c was not only closely associated with the risk of hypertension, but also positively correlated with SBP (26). Furthermore, Song et al. not only confirmed a strong correlation between HbA1c and the risk of hypertension in a Chinese population, but also unexpectedly found that it could also increase the risk of isolated systolic hypertension (27). Thus it can be seen that the relationship between HbA1c and the prevalence and incidence of hypertension has not reached a unified conclusion, and the causal relationship between HbA1c and hypertension has not been determined. What is encouraging is that our study found meaningful results that higher HbA1c was closely associated with a higher risk of hypertension, independent of traditional cardiovascular risk factors, including age, SBP, and BMI. In addition, The annual incidence of hypertension in this study was 10%, while the annual incidence of hypertension in the cohort study conducted by Lou et al. was 2.64% (28), and the probability in the study conducted by Heianza et al. was 2.29% (19). It can be seen that the incidence of hypertension in our study participants was higher than that in other studies, which was mainly related to the heterogeneity of the study population, and their study participants are mainly people without diabetes, which means that the cardiovascular metabolic risk of these people is relatively low, so the incidence of hypertension is relatively low.

In addition to hypertension, some studies have also shown that HbA1c is not only closely related to cardiovascular disease and poor cardiovascular outcomes (12–14, 22), but also inextricably related to all-cause mortality (14). These findings suggest that controlling HbA1c in the best range can not only reduce the incidence of diabetes, but also reduce the incidence of diabetic complications, cardiovascular disease morbidity, cardiovascular mortality and all-cause mortality, and further reduce the socio-economic burden and the health burden of the people, which is undoubtedly a great blessing for public health problems.

Additionally, not only the association between HbA1c and hypertension has not been agreed, but also the pathological mechanism of the harmful effects of higher HbA1c on hypertension is still unknown. There may be the following mechanisms involved in the pathogenic effect of HbA1c on hypertension. For example, higher HbA1c often reflects insulin resistance, and there is evidence that insulin resistance can promote the release of inflammatory factors, which in turn leads to endothelial dysfunction and the increase of sympathetic nerve tension, and may accelerate the reabsorption of sodium and water by renal tubules at the same time, eventually leading to the occurrence and development of hypertension (29–31). In addition, high HbA1c can reflect the state of continuously rising blood glucose, while persistently high glucose may induce the formation of advanced glycation end products, promote oxidative stress and activate protein kinase, thus damaging the stability and balance of endothelial cells and smooth muscle cells, leading to hypertension (32–35).

Despite our valuable results, there were several limitations that warrant discussion. First, although this was a prospective cohort study, it was still an observational study, so the causal link between HbA1c and hypertension was unknown in this population. Second, because the variable of physical activity was missing more in our study, we did not include this variable in our analysis and could not determine the extent of its effect on the association between HbA1c and hypertension. Third, due to the limitations of the data, we were unable to evaluate the effects of chronic kidney disease and hypothyroidism on the association between HbA1c with hypertension. Additionally, since HbA1c was measured only once at baseline, it was not possible to evaluate the impact of the trajectory of HbA1c change on hypertension. Besides, because our study lacked the variable of family history of hypertension, we were unable to evaluate the effect of family history of hypertension on the association between HbA1c and hypertension risk. And in this study, the annual incidence of hypertension was higher than that of other studies, so according to the epidemiological diagnostic criteria, the incidence of hypertension might not be so accurate. Finally, since all participants were from the CHNS, there was no systematic assessment of the causes of hypertension, so secondary hypertension could not be ruled out.

## 5 Conclusion

In this prospective cohort study from Chinese population, we found that there was a close linear positive correlation between HbA1c and the risk of hypertension, which not only further strengthened the close relationship between blood glucose fluctuation and the risk of hypertension, but also reminded us to pay attention not only to the traditional risk factors of hypertension, but also to the effect of blood glucose fluctuation on blood pressure level or hypertension.

## Data availability statement

The original contributions presented in the study are included in the article/supplementary material. Further inquiries can be directed to the corresponding authors.

## Ethics statement

The studies involving human participants were reviewed and approved by institutional review committees at the University of North Carolina at Chapel Hill and the National Institute of Nutrition and Food Safety, Chinese Center for Disease Control and Prevention. The patients/participants provided their written informed consent to participate in this study.

## Author contributions

XH conceived, designed the study. CQ contributed to initial data analysis and interpretation. XH and XG drafted the initial manuscript. CQ, FC, and CT revised the manuscript. FC and CT were the guarantor of this work and had full access to all the data in the study and take responsibility for its integrity and the accuracy of the data analysis. All authors read and approved the final manuscript.
